# Expression of MACC1 and c-Met in human gastric cancer and its clinical significance

**DOI:** 10.1186/1475-2867-13-121

**Published:** 2013-12-10

**Authors:** Tiankang Guo, Jingyu Yang, Jibin Yao, Yongbin Zhang, Mingxu Da, Yaoxing Duan

**Affiliations:** 1 Department of Surgical Oncology, Gansu Provincial Hospital, Lanzhou, 730000, PR, China; 2 Shandong Institute of Parasitic Diseases, Jining 272033, PR, China; 3 Department of Surgery, Ningxia Medical University, Yinchuan 750004, PR, China

**Keywords:** Gastric cancer, Metastasis associated with colon cancer1, c-Met, Peritoneal metastasis, Lymph node metastasis, Hepatic metastasis

## Abstract

**Background:**

Recent studies have suggested that the metastasis-associated colon cancer1 (MACC1) gene can promote tumor proliferation, invasion and metastasis through an upregulation of c-Met expression. However, its role in gastric cancer is controversial. Our study investigated expression of MACC1 and c-Met in gastric cancer, as well as correlated this with clinicopathological parameters.

**Methods:**

Expressions of MACC1 and c-Met protein in a sample of 98 gastric carcinoma and adjacent nontumorous tissues were detected by immunohistochemistry. Their relationships and correlations with clinicopathological features were analyzed.

**Results:**

The positive rates of MACC1 and c-Met protein in primary tumors were 61.22% and 59.18%, respectively. A significant correlation was found between expression of MACC1 and c-Met (P<0.05). Expression of the MACC1 protein in gastric cancer tissue was correlated with lymph node metastasis (χ^2^ = 10.555,P = 0.001), peritoneal metastasis (χ^2^ = 5.694, P = 0.017), and hepatic metastasis (χ^2^ = 4.540,P = 0.033), but not with age, gender, tumor size, location, clinical stage or the distant metastases (P>0.05).

**Conclusion:**

The positive rate of MACC1 protein expression was related to the protein expression of c-Met. Both had a correlation with the presence of peritoneal metastasis, lymph node metastasis and hepatic metastasis, all of which contribute to a poor prognosis for gastric cancer patients.

## Introduction

Gastric cancer is a common gastrointestinal malignancy. Although the incidence of gastric cancer has declined during the past years, it is still the fourth leading cause of cancer-related death. Gastric cancer is the second most frequent cause of cancer-associated death in malignant tumors that accounts for about 10.4% [1]. An analysis of the global incidence of cancer mortality showed that there are nearly 900,000 new cases and 700,000 cancer deaths in the world. The incidence of gastric cancer is significantly different in different countries and regions. Gastric cancer is the second most frequently diagnosed cause of cancer death in China [2]. Gastric cancer is a multifactorial disease with a complex interplay between genetics and both lifestyle and environmental factors, which consequently results in malignant transformation and progression of gastric cancer. Unfortunately, there has been no specific signature of gastric cancer gene expression reported to allow for patient-tailored therapy strategies. Accordingly, there is great demand to further identify novel oncogenes and clinically applicable molecular targets for the diagnosis and treatment of this disease.

Recent observations have suggested that the metastasis-associated colon cancer1 (MACC1) gene can promote tumor proliferation, invasion and metastasis, which is an independent prognostic indicator of recurrence and disease-free survival. The hepatocyte growth factor (HGF)/mesenchymal–epithelial transition factor (c-Met) pathway plays a key role in the carcinogenic pathway [3]. Hepatocyte growth factor (HGF) is a glycoprotein secreted by a variety of mesenchymal or tumor cells [4], which promote migration, invasion, wound healing and survival and suppress apoptosis by c-Met. Met transmits intracellular signals via the mitogen-activated protein kinase (MAPK) and PI3K–AKT pathways. MACC1 was reported to be elevated in various cancer tissues, including ovarian cancer [5], hepatocellular carcinoma [6], non-small cell lung cancer [7], and oral squamous cell carcinoma [8]. Recently, there have been reports demonstrating that MACC1 may be involved in the growth of blood vessels, lymphangiogenesis and metastasis of gastric cancer [9-11], but little is known regarding its role in gastric cancer development.

MACC1 may be involved in the development of gastric cancer through the HGF/c-Met pathway, or as an independent factor in the gastric process, or through other pathways. MACC1 may become a new molecular marker and target for the diagnosis and treatment of gastric cancer proliferation and metastasis. In the present study, we investigated MACC1 and c-Met expression by immunohistochemistry and real-time polymerase chain reaction (RT-PCR) and analyzed the relationship, as well as correlating it with clinicopathological parameters and their clinical significance.

## Materials and methods

### Patients and tumor specimens

Ninety-eight cases of gastric cancer tissues and adjacent noncancerous mucosa were collected at Gansu Provincial Hospital from Apr 2006 to Feb 2007. Tissue samples for diagnostic purposes were obtained with the consent of each patient. All tumor specimens and corresponding normal tissues were fixed in 10% buffered formalin, embedded in paraffin, and then made into continuous 4 μm tissue sections for immunohistochemical examination. All patients had been histologically diagnosed without preoperative radiotherapy, chemotherapy or other anti-cancer therapy. All of the cases received postoperative adjuvant chemotherapy, and all specimens were pathologically confirmed. The study group consisted of 98 patients, 69 males and 29 females, aged 17 to 79 years, the average age (65.34 ± 13.40) years, and the median age 58 years. The staging of gastric cancer was according to the American Joint Committee on Cancer (ACJJ, 7th edition) (Table 1).

**Table 1:** Correlation of clinicopathological parameters with MACC1 and c-Met expression in gastric cancer

**Clinicopathological features**	**No. of cases**	**MACC1**			**c-Met**		
		**(+)**	**(-)**	***P***	**(+)**	**(-)**	***P***
Age(years)							
<55	41	24	17	0.643	25	16	0.760
≥55	57	36	21		33	24	
Gender							
Male	69	42	27	0.911	41	28	0.941
Female	29	18	11		17	12	
Maximal tumor size(cm)							
<5	54	34	20	0.696	35	19	0.209
≥5	44	26	18		23	21	
Tumor location							
Cardia, fundic, body	43	30	13	0.125	29	14	0.141
Antral	55	30	25		29	26	
Histology							
Well differentiated	39	20	19	0.101	20	19	0.196
Poorly differentiated	59	40	19		38	21	
Lymphnode metastasis							
Yes	72	51	21	0.001*	47	25	0.041*
No	26	9	17		11	15	
Peritoneal dissemination							
Yes	26	21	5	0.017*	20	6	0.032*
No	72	39	33		38	34	
Hepatic metastasis							
Yes	18	15	3	0.033*	15	3	0.021*
No	80	45	35		43	37	
TNM stage							
I,II	43	23	20	0.165	22	21	0.153
III,IV	55	37	18		36	19	
MACC1							
(+)					43	17	0.002*
(-)					15	23	
Odds ratio							3.878

*P <0.05.

Rabbit anti-human MACC1 and c-Met antibodies were purchased from Beijing Biosynthesis Biotechnology Co., Ltd., SP immunohistochemical detection kit. DAB chromogenic kit was purchased from Beijing Zhongshan Golden Bridge Biotechnology Co., Ltd.

### Immunohistochemistry (IHC)

Gastric cancer tissues paraffin sections were placed in citrate buffer (pH 6.0) for antigen retrieval. The negative control antibody was replaced by PBS. The positive controls were lung adenocarcinoma specimens expressing MACC1, and c-Met expression of hepatocellular carcinoma. The procedure was in accordance with the immunohistochemical SP detection kit instructions. The diluted density of rabbit anti-human MACC1 antibody was 1:100 and c-Met antibody was 1:150. MACC1 and c-Met protein positive products were mainly localized in the cytoplasm. A small number of nuclear membranes were colored pale-brown with a diffuse distribution.

### Evaluation of IHC staining

Positive results were judged by semi-quantitative points [9]. The staining intensity score was 0 (negative), 1 (weak), 2 (medium), and 3 (strong). The integral of the rate of positive cells was 0(0%), 1 (1-25%), 2 (26-50%), 3 (51-75%), and 4 (76-100%). The proportional score and the intensity score were then added to obtain a total score. A score ≥ 3 was considered to be a positive expression.

### Statistics

SPSS 16.0 statistical software was used for analysis, and count data used the *χ*^2^ test. The Kaplan-Meier method was used to estimate cumulative survival as a function of time, and survival differences were analyzed with the log-rank test. The inspection level *α* = 0.05.

## Results

### MACC1 and c-Met protein expression in gastric cancer tissues and adjacent normal tissue

**Figure 1:**
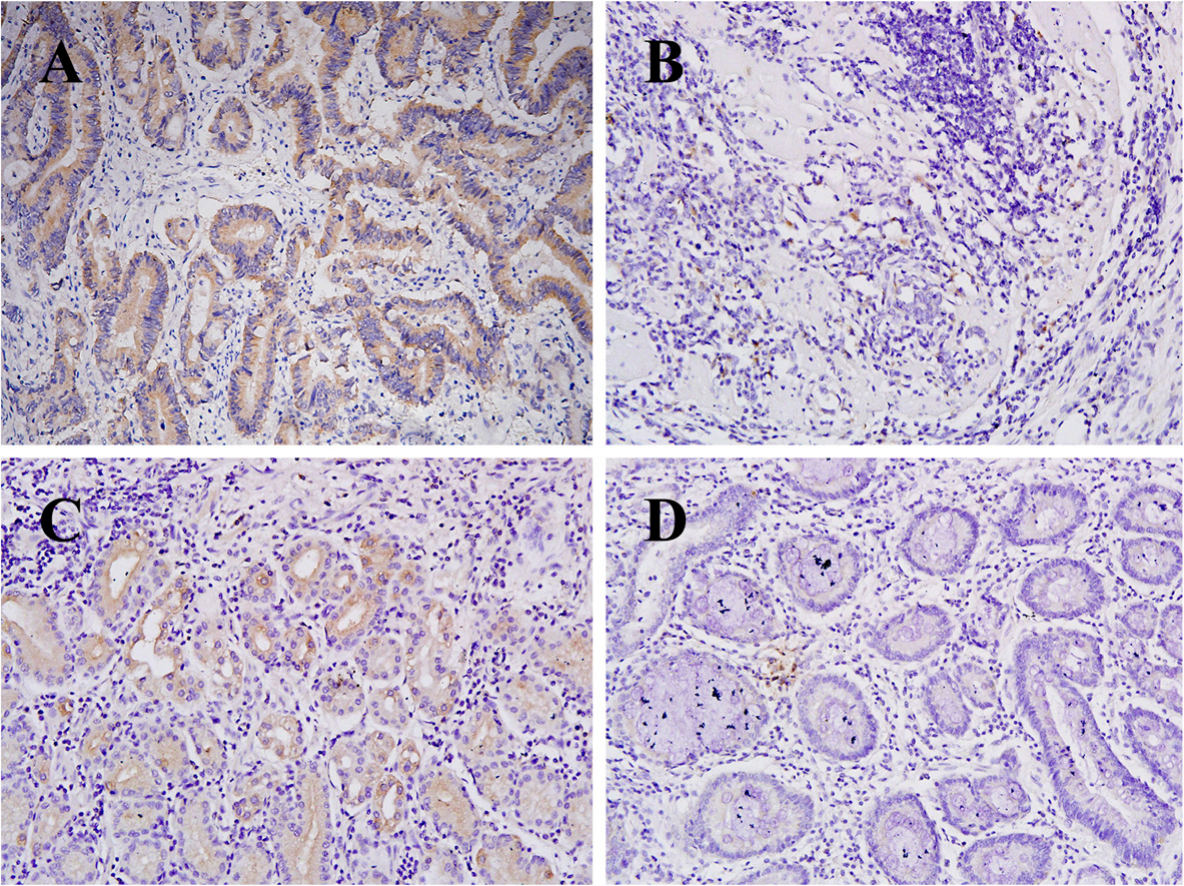
**Representative IHC staining (SP × 100). A**: Positive labeling for MACC1 with brown stained cytoplasm is shown in primary gastric carcinoma. **B**: Negative staining for MACC1 is shown in primary gastric carcinoma. **C**: MACC1 protein expression in adjacent normal tissues **D**: Negative expression in adjacent noncancerous mucosa.

**Figure 2:**
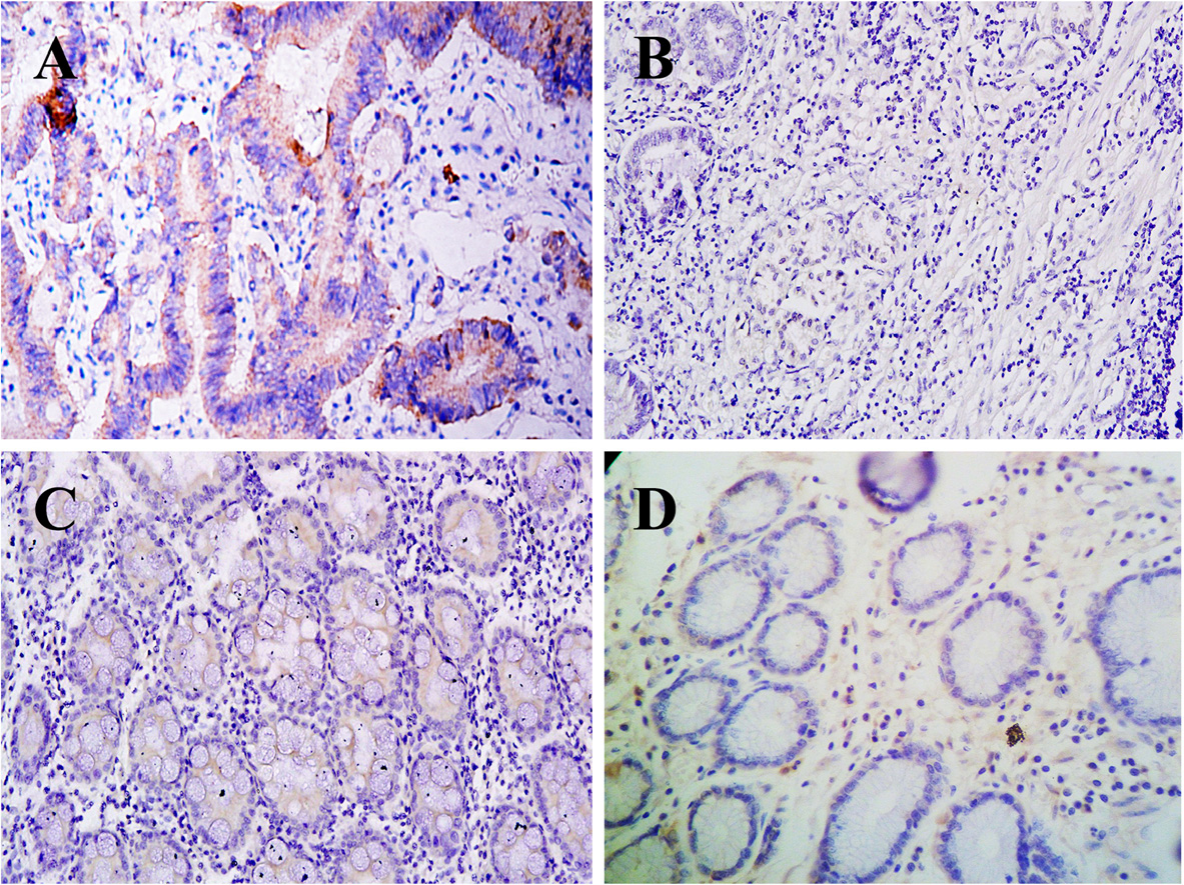
**C-Met protein expression determined by IHC staining in gastric carcinoma and adjacent normal tissues (SP × 100). A**: c-Met protein expression in gastric cancer. **B**: Negative expression in gastric cancer. **C**: Positive staining for c-Met in adjacent normal mucosa. **D**: Negative staining for c-Met protein.

Immunohistochemical results showed that the positive rate of MACC1 in the 98 tumor tissues was 61.22% (60/98). It was significantly higher than in the corresponding normal tissues (9.18%, 9/98) and the difference was statistically significant (*χ*^*2*^ = 58.176, P<0.05) (Figure 1). The positive rate of c-Met in the 98 tumor tissues was 59.18% (58/98). It was significantly higher than in the corresponding normal tissues (10.20%, 10/98) and the difference was statistically significant (*χ*^*2*^ = 51.882, P<0.05) (Figure 2).

### Correlation of clinicopathological parameters with MACC1 and c-Met Expression

Immunohistochemical expression of MACC1 in gastric cancer tissues and the statistical analysis of clinical pathological data of patients showed that there was a close relationship between MACC1 which was highly expressed in tumors and lymph metastasis, peritoneal metastasis and hepatic metastasis. *P* values were 0.001, 0.017, 0.033, 0.001 respectively and the difference was statistically significant (Table 1). There was no association with age, gender, tumor size, location, clinical stage or the degree of histological differentiation (*P*>0.05). Expression of c-Met protein in the gastric cancer tissue was correlated with lymph node metastasis (*χ*^*2*^ = 4.172, *P =* 0.041), peritoneal metastasis (*χ*^*2*^ = 4.610, *P =* 0.032), and hepatic metastasis (*χ*^*2*^ = 5.323, *P =* 0.021), but not with age, gender, tumor size, location, clinical stage or the degree of histological differentiation (*P*>0.05).

### MACC1 and c-Met expression correlated with patient survival in gastric cancer

**Figure 3:**
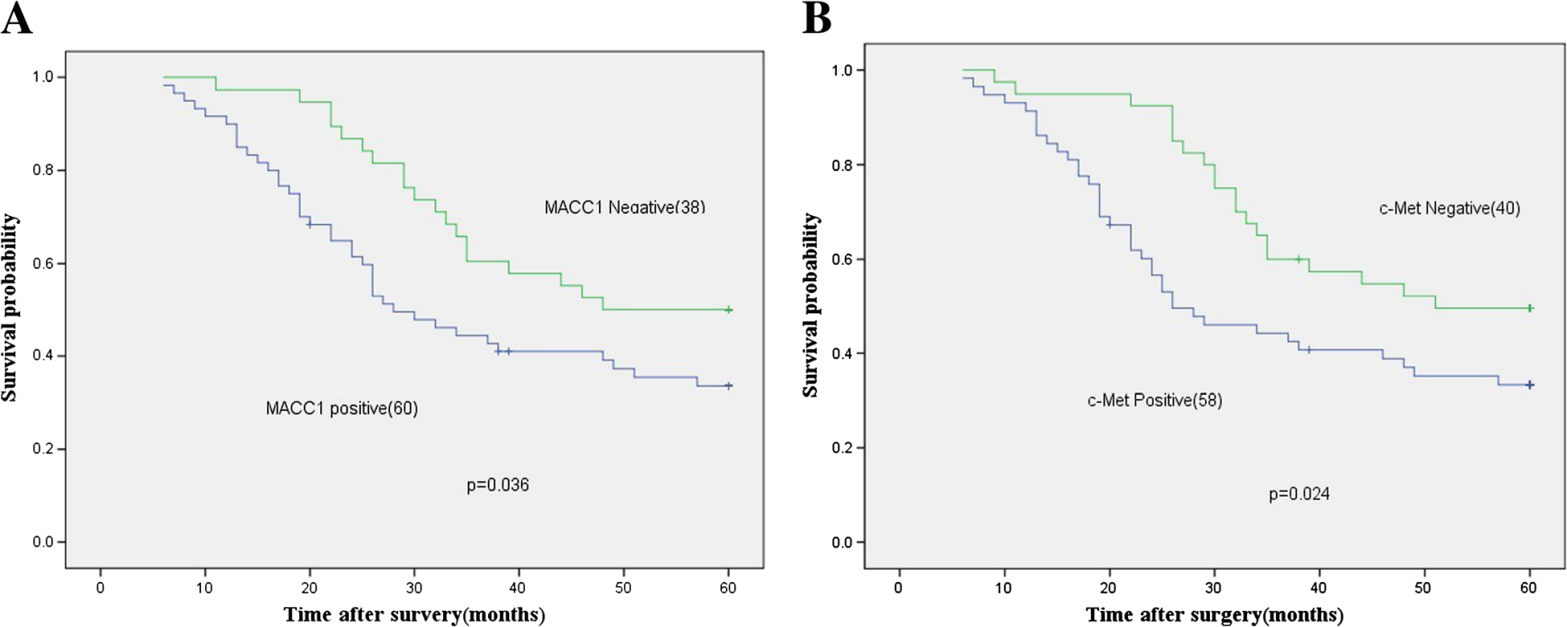
**Kaplan-Meier survival curves of patients with gastric cancer according to MACC1 (A) and c-Met (B) protein expression.** Correlation between overall survival of the patients and MACC1 expression was found to be statistically significant (log rank *p =* 0.036) as well as that between survival and c-Met expression (log rank *p =* 0.0024).

MACC1 protein was closely related to expression of c-Met in gastric cancer tissues, *P* = 0.002 (Table 1). As shown in Figure 3, the survival rate of MACC1 negative patients was significantly higher than in the positive expression group (*χ*^*2*^ = 4.386, *P* = 0.036). However, the survival rates were significantly different between the c-Met protein positive and negative groups of patients (*χ*^*2*^ = 5.072, *P* = 0.024).

## Discussion

Recently, Stein et al. [3] identified the metastasis associated with the colon cancer 1 (MACC1) gene by genome-wide expression analysis in primary tumors, metastases and normal mucosa of subjects with colon cancer. They also observed that MACC1 mRNA expression in colorectal carcinoma might be an independent prognostic indicator of metastasis formation and disease-free survival. The MACC1 gene is located on human chromosome 7 (7p21.1), contains seven exons and six introns, and the effective coding region of the gene is constituted of 2559 nucleotides. MACC1 encodes a putative protein of 852 amino acids. The main structure can be divided into four regions: the ZU5, SH3, and the two C-terminal hydroxyl death domains (DD).

Subsequent study found that there is a close relationship between MACC1 and proliferation, metastasis and recurrence of a variety of tumors. Previous studies have demonstrated that overexpression of MACC1 in lung adenocarcinoma is an independent risk factor for recurrence, and is closely related to the survival rate for patients [12-14]. MACC1 expression is significantly related to vascular invasion and alpha-fetoprotein level, which might serve as a novel prognostic marker in hepatocellular carcinoma [15]. The positive rate of MACC1 protein expression is related to the protein expression c-Met as shown by QRT-PCR in primary hepatocellular carcinoma and is closely related to recurrence and disease-free survival. Furthermore, patients with tumors expressing MACC1 at the TNM Ι stage have a poorer prognosis compared with those with expression-negative tumors [16]. MACC1 expression in ovarian cancer was significantly higher in primary tumors than in normal tissues. We found that high levels of MACC1 often correlated with enhanced lymph node metastasis, distant metastasis and the clinicopathological stage [17]. MACC1 specific small hairpin RNA (shRNA) expression plasmids were constructed and transfected into OVCAR-3 cells, which decreased expression of MACC1 and resulted in significant inhibition of cell proliferation, migration and invasion, with obvious enhancement of apoptosis [18].

Expression of the MACC1 protein through up-regulation of c-Met is by action on the Sp1 sites, which is the c-Met startup sequence that enhances the cascade effect of HGF/c-Met signaling pathways [19]. Our results suggest that MACC1 protein expression is consistent with c-Met expression in gastric cancer specimens, which was up-regulated along with the progression of gastric cancer. These results suggest that MACC1 may act as a new parameter for predicting poor prognosis of gastric cancer. Thus, the current findings provide evidence that positive expressions of MACC1 and c-Met in gastric cancer are significantly higher in primary tumors which developed lymph node metastasis, compared to those with no metastasis. It indicates that the MACC1 protein can promote lymph node metastasis through the HGF/c-Met pathway. Research has shown that the HGF/c-Met pathway is related to gastric cancer lymphangiogenesis [20], which directly or indirectly promotes lymph node metastasis through the VEGF-C/VEGF-D/VEGFR-3 axis [21,22]. Taken together, these results revealed that the MACC1 protein can forecast lymph node metastasis, but the specific mechanism of how it is involved in lymph node metastasis needs further examination.

Peritoneal metastasis is a common sign of advanced tumor stage, tumor progression, or disease recurrence in patients with gastric cancer [23]. Our study shows that positive protein expression of c-Met and MACC1 seems to be related to the peritoneal metastasis of gastric cancer, which is consistent with Shirahata, A [24] related conclusions. MACC1 expression might be an indicator for peritoneal dissemination of gastric cancer, but its specific mechanism is unclear. Toiyama, Y. et. al [25] demonstrated that the HGF/c-Met pathway induces epithelial mesencymal transition (EMT), which has the potential to promote peritoneal dissemination in gastric cancer. Epithelial–mesenchymal transition (EMT) allows epithelial cells to have mesenchymal-cell characteristics and to acquire the more invasive characteristics of mesenchymal cells, which is also involved in metastasis [26]. The EMT process of tumor development can facilitate migration and invasion of epithelial tumor cells [27].

Another clinical result showed a strong correlation between expression of HGF/c-Met and abnormal expression of E-cadherin in tumor cells, which can be viewed as both a cause and effect of EMT [20]. We speculate that the MACC1 protein may induce the process of EMT in gastric cancer cells through the HGF/c-Met pathway, and consequently influences the peritoneal metastasis of gastric cancer. Therefore, determination of this mechanism might provide clinically useful information for peritoneal dissemination and prognosis in patients with gastric cancer.

The five-year survival rate for gastric cancer patients with liver metastases remains less than 5% [28], and there are still no effective means of prevention and treatment [29]. The present study confirmed that c-Met was closely related to liver metastasis of gastric cancer which is consistent with the literature [9]. Also, high MACC1 protein expression was significantly associated with liver metastasis of gastric cancer. It suggested that MACC1 might play an important role in the progress of liver metastases from gastric carcinoma. MACC1 may be used as a new predictive molecular marker of liver metastasis of gastric cancer, and down-regulation of MACC1 may provide a novel method for blocking the process of liver metastasis from gastric cancer. More experiments are needed to verify these hypotheses.

Our results which are consistent with the majority of cancer research suggest that MACC1 and c-Met are the poor prognostic indicators of gastric cancer, which are different from Ge Sh et al. [30] findings. When Stein elaborated the mechanism of MACC1 protein, she reported that MACC1 mutants lacking the SH3 domain or prolin-rich sequence abrogated the binding of MACC1 to the Met promoter [31]. More research on this is needed in the future.

In conclusion, it was found that expression of MACC1 correlated with c-Met expression and that both of them correlated with the presence of lymph node metastasis, peritoneal metastasis, and hepatic metastasis in gastric carcinoma. These results suggest that MACC1 and c-Met may serve as parameters for the prognostic prediction of gastric cancer. Furthermore, the specific mechanisms that are involved in the progression of gastric cancer needs further examination.

## Competing interest

The authors declare that they have no competing interest.

## Authors’ contribution

All authors read and approved the final manuscript.
